# Capturing Requirements for a Data Annotation Tool for Intensive Care: Experimental User-Centered Design Study

**DOI:** 10.2196/56880

**Published:** 2025-02-05

**Authors:** Marceli Wac, Raul Santos-Rodriguez, Chris McWilliams, Christopher Bourdeaux

**Affiliations:** 1 Faculty of Engineering University of Bristol Bristol United Kingdom; 2 University Hospitals Bristol and Weston NHS Foundation Trust Bristol United Kingdom

**Keywords:** ICU, intensive care, machine learning, data annotation, data labeling, annotation software, capturing software requirements

## Abstract

**Background:**

Increasing use of computational methods in health care provides opportunities to address previously unsolvable problems. Machine learning techniques applied to routinely collected data can enhance clinical tools and improve patient outcomes, but their effective deployment comes with significant challenges. While some tasks can be addressed by training machine learning models directly on the collected data, more complex problems require additional input in the form of data annotations. Data annotation is a complex and time-consuming problem that requires domain expertise and frequently, technical proficiency. With clinicians’ time being an extremely limited resource, existing tools fail to provide an effective workflow for deployment in health care.

**Objective:**

This paper investigates the approach of intensive care unit staff to the task of data annotation. Specifically, it aims to (1) understand how clinicians approach data annotation and (2) capture the requirements for a digital annotation tool for the health care setting.

**Methods:**

We conducted an experimental activity involving annotation of the printed excerpts of real time-series admission data with 7 intensive care unit clinicians. Each participant annotated an identical set of admissions with the periods of weaning from mechanical ventilation during a single 45-minute workshop. Participants were observed during task completion and their actions were analyzed within Norman’s Interaction Cycle model to identify the software requirements.

**Results:**

Clinicians followed a cyclic process of investigation, annotation, data reevaluation, and label refinement. Variety of techniques were used to investigate data and create annotations. We identified 11 requirements for the digital tool across 4 domains: annotation of individual admissions (n=5), semiautomated annotation (n=3), operational constraints (n=2), and use of labels in machine learning (n=1).

**Conclusions:**

Effective data annotation in a clinical setting relies on flexibility in analysis and label creation and workflow continuity across multiple admissions. There is a need to ensure a seamless transition between data investigation, annotation, and refinement of the labels.

## Introduction

### Background

Artificial intelligence (AI) is a field concerned with leveraging computers to mimic human cognitive functions, such as problem-solving and decision-making [1,2]. Depending on the task, this process can use a variety of methods and take many forms ranging from relatively simple rule-based expert systems (following if-then pathways) through regression (modeling the relation between different variables to predict their values) and clustering (grouping objects with similar properties together) to more complex systems such as artificial neural networks (computing systems imitating the anatomy of human brains capable of modeling nonlinear processes) [1,3,4]. Particularly complex problems may require solutions that cannot be achieved by traditional approaches, such as preprograming the desired behavior. Instead, techniques such as machine learning (ML) form a subset of AI in which the algorithms are used to analyze large amounts of data to derive a method for computing a solution [5]. In ML, the practice of arriving at a solution is called “learning” (or training), and the produced output is known as the ML model. The heavy reliance on data in ML highlights the importance of ensuring the appropriate quantity and quality of data and signifies its impact on the effectiveness of the created model [6]. The continuously increasing popularity of ML and its rapid adoption rate in life sciences suggest that AI is at the forefront of bringing innovation to health care [7,8].

Intensive care units (ICUs) are busy and complex health care environments where critically ill patients frequently require continuous monitoring and multiple-organ support. To provide care for those patients, clinicians working in the ICUs use a broad range of medical devices, such as ventilators, monitoring devices, and intravenous pumps and lines among many others. The information captured by those devices, as well as that entered by the ICU staff, is collected and collated in a clinical information system, which enables health practitioners to access large quantities of data routinely required as part of their job. This data-rich nature and the direct influence of data on the provision of care provide a tremendous opportunity for the deployment of ML models in health care and in particular ICUs [9].

**Figure 1:**
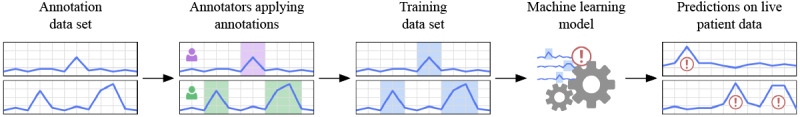
Annotating data can provide additional information and context necessary to train machine learning models.

While the data gathered in the ICUs can often be used directly, for example, to present the correlation between different vital signs and the patient prognosis, complex tasks that rely heavily on clinical experience and expertise may require human involvement to provide further guidance [10]. This guidance, most frequently referred to as labels or annotations [11], can deliver additional information or context to the existing data. An example of such a label could be a “yes” or “no” indicator of whether a patient is ready for discharge, or the type and size of tumor present on a radiograph. With this further knowledge, ML models can take advantage of the human experience to tackle complex problems and deliver solutions that could not be inferred from raw data alone [12] (Figure 1).

While the large volumes of data available in intensive care offer significant opportunities, working with these data also presents a unique set of challenges, as the effort required to annotate the dataset increases proportionally to its size [13]. Furthermore, the complex nature of the health care data and the fact that applying a single label can require clinicians to look at multiple parameters, patient history, and laboratory results make the annotation task highly labor-intensive. With the clinician’s time being a remarkably valuable resource, ensuring the effectiveness of the data annotation workflow is paramount. The lack of an annotation system tailored to the unique nature of the health care data (eg, accessing a subset of relevant variables from a list of potentially hundreds of parameters [14]) further complicates this problem.

In addition to the difficulties associated with working within clinical settings, it is equally important to consider the inherent challenges of data annotation in their own right. These include the need to account for multiple different types of bias that could be introduced throughout the process [15]. For example, the annotators themselves may have their own preferences and preconceptions that will guide the approach they assume when annotating data. This could stem from the amount and diversity of their clinical experience in a context specific to the annotation task, such as the familiarity with the clinical problem, the specific population within which they have treated it, or even the specific tools and methods they have used in the past. Similarly, by annotating historical data that span the entire duration of the admissions, annotators may create labels with an inherent temporal or selection bias. Alternatively, annotators tasked with creating specific annotations may focus on a subset of the admission where they would expect to create the annotation, such as reliance on medication, which may decrease toward the end of the admission but could equally change throughout its overall course.

Current literature on the applications of computational methods within health care highlights data annotation as a primary bottleneck in the ML pipeline [16]. This effect is attributed to the need for domain-specific knowledge and therefore access the expert population, as well as the considerable investment of their time [16-18]. Because of this, existing methods aim to reduce the number of required annotations required for positive results by using a variety of techniques including active learning [17], machine-assisted annotation [19], or synthetic data generation [20]. Nevertheless, the majority of studies continue to rely on human annotations in the cases where labels cannot be easily derived, or are inadequately documented in structured data [16]. Designing a tool for the annotation of large clinical datasets is therefore a problem that needs to be approached carefully. The expert nature of the annotators means that both their numbers and time are limited and are therefore resources that need to be used efficiently [16]. Furthermore, clinical datasets frequently aggregate data spanning several years, resulting in a volume of data that is infeasible for manual annotation, suggesting a need for a semiautomated approach that could scale up to an entire dataset with limited input from the domain experts.

Involving end users in the design process of new tools prior to their development is an important aspect of designing effective software [21]. It minimizes the risk of creating a system that is inefficient and helps ensure that the developed solution meets users’ expectations and requirements [22,23]. In the context of the data annotation software used by experts in intensive care, this participatory design is especially critical, as each of the variety of roles (eg, junior doctors, doctors, nurses, and consultants) can generate a unique set of requirements. Furthermore, while the staff working in the ICUs are experts in the medical domain trained to treat patients, extracting their knowledge through data annotation is an entirely different process. For this reason, it is crucial to deploy a strategy that will ensure that the design of the tool follows a structured and systematic approach that can capture a wide variety of perspectives from its end users while also accounting for the needs and priorities of the data science experts who will use the created annotations.

### Objectives

The primary objective of the research is to establish a set of criteria for the design of a data annotation tool that can be used effectively by clinicians to annotate time-series datasets from intensive care. To achieve this goal under the limitations of working within the health care setting, the system needs to account for limited access to the annotators and large volumes of data. Finally, to ensure the efficiency of the process, the subtleties of how clinicians approach the problem of data annotation need to be understood and accounted for in the design. The overarching goal is, therefore, to gather requirements for a data annotation platform that is purpose-built for the intensive care data and clinicians and, as such, one that facilitates an efficient annotation workflow in that domain.

## Methods

### Study Design

To understand how clinicians approach and reason about the data annotation process, we conducted an experimental study that involved members of the clinical staff from the ICU manually annotating excerpts of time-series data printed on paper. Observations taken during task completion served as a basis for analysis and were used to derive the requirements for the data annotation software. This choice of methodology was influenced by constraints associated with working within the clinical setting, such as the high demand and short supply of participants’ time, and the direct relationship between the task completion and the requirements for the digital tool, which allowed for a natural and unobstructed data collection.

### Participant Recruitment

Our participant recruitment followed a mixture of convenience and snowball sampling comprising an invitation email sent to the staff working in the ICU who had prior experience with mechanical ventilation treatment and signposting through the internal network at our research site—University Hospitals Bristol and Weston NHS Foundation Trust. This recruitment process yielded a sample size of 7 participants across 2 distinct job roles (6 junior doctors and 1 doctor), which provided limited feasibility for suitable analysis of the interrole differences in approach to annotation but was sufficiently rich for establishing the software requirements for the tool we set out to design. Comparative sample sizes can be observed in similar research in the field [24-26]. Before data collection, the annotation task was piloted with a clinical member of our team experienced in mechanical ventilation treatment, as well as 2 other members of our team from the ML background. The insights collected from the pilot activity allowed us to refine the set of clinical parameters used throughout the activity, devise the inclusion criteria for the underlying admissions, and diversify the data extracts to form a sample representative of a typical dataset. Our research site had a preestablished research partnership with the University of Bristol and a technologically enhanced ICU with widespread adoption of digital systems.

### Theoretical Framework

To analyze the participants’ actions performed during the simulated activity, we used a framework that served as a basis for characterizing the data annotation process and identifying the challenges and opportunities associated with performing the task. Our framework followed the Norman’s Interaction Cycle (NIC) model which assumes that the interaction is a process of evaluation and execution between the user and the technology [27]. It outlines a 7-step process that begins at the goal and, through evaluation of available means and strategies, as well as the execution of these strategies, leads to achieving that goal in practice. The framework emphasizes the breakdown of the preproduction stage into planning (formulating the problem that needs to be solved), specification (outlining the strategies that can be used to tackle the problem), and performance (specifying actions that need to be taken to deploy the strategy). Furthermore, it highlights the importance of presenting the user with feedback over 3 separate stages including perception (tracking the outcome of performed actions), interpretation (analyzing the effect of the outcome), and comparison (evaluating whether their actions resulted in achieving the goal) [27]. Finally, the model captures the disparities between user intentions and actions permitted by the technology (gulf of execution) and the effort required to correctly interpret the results of their actions with regard to the desired outcome (gulf of evaluation) [27,28].

### Data Collection

**Figure 2:**
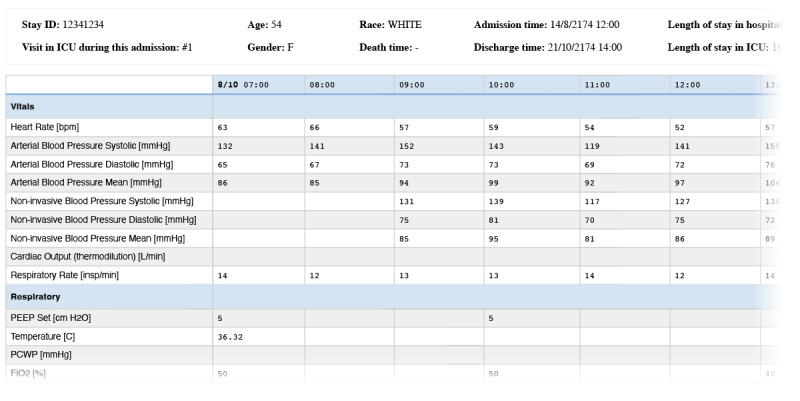
The provided excerpts of time series data were formatted to resemble the interfaces of the clinical information system present at the research site (the depicted table contains data for illustrative purposes only and is trimmed for conciseness). FiO2: fraction of inspired oxygen; ICU: intensive care unit; PCWP: pulmonary capillary wedge pressure; PEEP: positive end-expiratory pressure.

**Figure 3:**
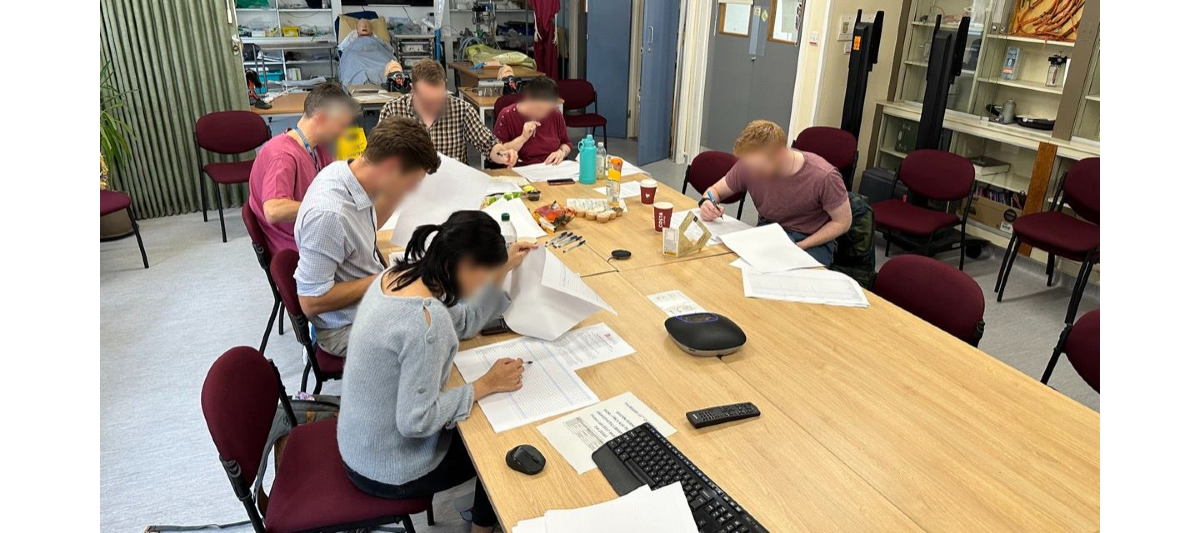
Participants annotated data in a variety of ways using pen and paper during the workshop held in the intensive care unit.

To facilitate data collection, we simulated a data annotation activity involving manual annotation of real intensive care patient data using pens and highlighter pens on excerpts of data printed on paper sheets. The data were formatted to resemble the clinical information system present at the research site and displayed patient demographics in addition to a table with time-series data. Each column of the table corresponded to an hour of the day and each row corresponded to a specific parameter; the table’s cells contained the readings of a parameter for a given hour (Figure 2). The outline brief for the activity prompted participants to create annotations, allowing them to create multiple annotations for a single admission. Crucially, no additional instructions were provided regarding the annotation format. Before the data collection, the activity was piloted internally with an experienced intensive care consultant (CB) and 2 ML experts (RSR and CMW), which helped us establish inclusion criteria for the selected admissions, broaden the characteristics of our admission sample, and refine the included parameters. The activity took place as a single in-person workshop, lasting approximately 45 minutes, during which each of the 7 participants annotated the same set of 5 unique admissions (Figure 3).

Participants were observed for the duration of task completion by a single observer who took field notes throughout the entire activity. These field notes captured the interactions of the participants with the annotation tasks and described the actions taken when annotating specific admissions, how multiple admissions were annotated, and questions asked by the participants during the task. The collected notes were then aggregated across the different stages of NIC and the annotated printouts were collected and used to supplement the analysis of the methods used by the participants to label the data.

### Task Selection

Capturing data labels in a manner representative of the general annotation process relied on selecting an appropriate task. On the one hand, the desired label could not be trivial and needed to pose a significant challenge, complex enough to prompt an in-depth analysis of the data. On the other hand, this task also needed to fall within the subset of domain knowledge that participants were familiar with and could therefore solve with a relative degree of confidence. Annotating weaning from mechanical ventilation satisfies both of these criteria due to its challenging nature and the relative bias of the person who performs it. The process of weaning can be characterized by great variability in practice [29], in which both the timing of when the weaning begins and the method in which it is delivered largely depend on the clinician in charge of the treatment [29,30]. Together with the lack of personalized guidelines [31] and a wide variety of ways in which patients can be weaned, mechanical ventilation weaning constitutes a label that is difficult to derive from data and requires domain expertise, which makes it suitable for this task. While in the context of mechanical ventilation the term “weaning” comes with an inherent ambiguity due to a number of conflicting definitions [32-36], for the purpose of this activity it was explicitly defined as “the reduction of support delivered by the mechanical ventilator with an end goal of extubation.” Consequently, the activity brief prompted participants to annotate periods during which mechanical ventilation weaning takes place and allowed multiple annotations for any individual admission.

### Admission Data

The data used in the activity came from a deidentified intensive care dataset called “Medical Information Mart for Intensive Care IV version 2.0” [37]. The eligibility criteria required that subjects were at least 18 years of age at the time of admission, had undergone an invasive mechanical ventilation treatment that lasted for a minimum of 24 hours, their stay in the ICU lasted for a minimum of 4 days, and that their stay did not end with death, including up to 48 hours after discharge. The time-series parameters used in the data extract were limited to those relevant to mechanical ventilation weaning and were selected by 2 independent clinicians working in the ICU. The data were extracted using a structured query language script run on the PostgreSQL installation of Medical Information Mart for Intensive Care IV version 2.0 with the concept tables computed [38]. The script aggregated the selected parameters on an hourly basis for each eligible admission and limited it to the range surrounding the period of mechanical ventilation treatment.

### Ethical Considerations

This work was approved by the Faculty of Engineering Research ethics committee at the University of Bristol (case 2022-150, research ethics committee reference 22/HRA/2166). Information regarding the study was circulated with the invited participants electronically alongside the recruitment email. Informed consent was captured electronically using digitally signed consent forms prior to enrollment in the study. Data obtained from this study were deidentified and stored securely in an encrypted database. The access to the database was password-protected and only the research team had access to these data. All participants were able to quit without any explanation at any point during the study. Participation in the study was voluntary, and no financial or other form of compensation was provided to the participants.

## Results

### Data Annotation Analysis

During the simulated data annotation activity, we observed participants following a series of discrete steps when annotating data. To understand how these actions fit into the annotation process, we analyzed the observations in the context of NIC and aggregated them into the distinct phases of the model.

#### Planning

Following the distribution of the printout sheets containing the admission data, participants began the process of familiarizing themselves with the data. This stage involved selecting a single excerpt from the available admissions, analyzing the patient demographics, and browsing through the time-series data. During the process, participants frequently cycled through the entire length of the time-series data from the initial admission until discharge and back.

#### Specifying

Upon familiarization with the specific admission, participants began searching for individual points in time suggestive of the weaning taking place. Some participants preferred to start with the end of treatment, where weaning led to extubation, while others analyzed the data to find the points in time when weaning began. This involved focusing on specific parameters (as suggested by following the specific rows with the pen tip) while browsing through the time-series data across different columns.

#### Performing

Upon finding the thresholds for the start or end of weaning, the participants annotated the printed sheets directly. To create the labels, a variety of techniques were used, which differed both between the individual annotators and the distinct admissions. These included circling the start and end dates in the header row of corresponding columns, drawing vertical lines on the edges of the columns to mark the start and end periods, and drawing a box around the portion of the dataset spanning the duration of the label. Similarly to the analysis process, some participants preferred to first annotate the end of the weaning process, rather than its start. In some cases, participants also provided additional information in the form of written comments that justified the created label (eg, “mode of ventilation changed from A to B” or “delivered oxygen reduced—indicative of weaning”) and underlined values within cells of parameters that prompted annotation.

#### Perceiving

Following the act of annotating the data, participants frequently verified their labels. This was expressed by browsing through the time-series data again and ensuring that the start and end dates corresponded to the parameter readings that suggested the annotation in the first place.

#### Interpreting

In some cases, participants derived additional insights from the data when investigating them with their labels already present. These insights frequently manifested in the form of retracing the admission data over the span of the created label, which resulted in a reevaluation of the created label and its underlying data.

#### Comparing

Finally, participants who identified the need for amendments returned to the planning stage to repeat the annotation process, whereas those satisfied with their annotations frequently sought feedback from the activity facilitator on the desired annotation count and the next steps involved in the process.

**Figure 4:**
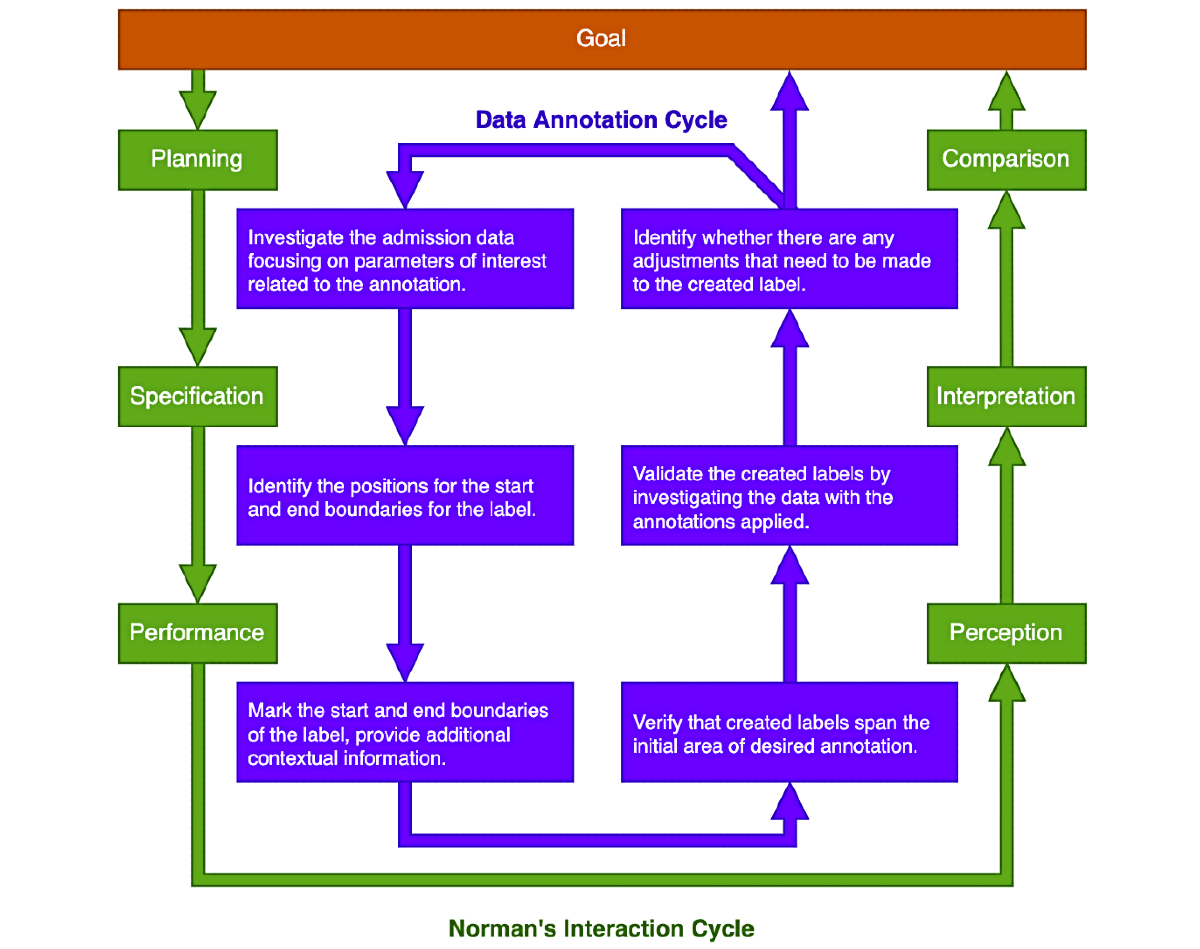
Data annotation followed a cyclic process involving investigation of data, label creation, reevaluation of data with labels applied, and refinement of created annotations.

Crucially, by contextualizing the annotation process within the NIC model we identified that the overall approach to annotation followed a cyclic process of investigation of the data, creation of the labels, reevaluation of the data with the labels applied, and refinement of previously created labels (Figure 4).

### User Requirements

Observations from the workshops captured key characteristics of participant interactions with the task, forming a basis for establishing the functional requirements of the digital tool. Coupled with the operational constraints of data annotation in clinical settings and the specific needs for future label use in the ML context, these characteristics informed the user requirements for the digital annotation tool.

#### Annotation of Individual Admissions

Familiarization with admission data during the planning and specification stages suggested that the primary goal of participants at the early stage of the process was to obtain a high-level overview of the patient’s characteristics and their stay in the ICU. This was supported by the frequent cycling through different parts of the admission, allowing participants to establish a broad timeline of events and the overall treatment trajectory without focusing on the minutia of its delivery. The interface of the tool should allow end users to conduct a similar analysis digitally, incorporating both the patient demographics and the time-series data.

Once familiarized with the admission data, participants shifted their focus to a more task-oriented analysis by narrowing the list of parameters to a subset significant for the task, suggesting different relevance of specific parameters to the target label. Furthermore, the fact that in certain cases participants found it easier to first search for the end of weaning, rather than its start, suggests that their confidence in the accuracy of the created label, its precise start or end, might be different across annotations. Similarly, each of the participants displayed different preferences for creating annotations favoring first marking either the start of the weaning process or its end. Despite this, they all shared a common set of actions of marking the beginning and end of the label, even when the order of these operations was not the same. To make the annotation process as unobtrusive as possible, the digital data annotation tool should therefore allow flexibility in not only how the data are viewed and analyzed but also how the annotations are being created. As such, the interface needs to display a continuous dataset without constraining it to a subset of time or parameters and allow the end users to freely mark the boundaries of the label in the order of their preference.

Following the creation of a label, participants frequently continued to investigate the underlying data and further adjusted the labels, indicating the need to reflect on their immediate performance and refine the annotations. Adjusting labels upon their reevaluation indicates that when the data are viewed with the annotations applied, the annotator’s perception of these data changes allowing for additional insights to be derived. As such, the digital tool should allow for the display of the created annotations on top of the data and facilitate a similar ability to adjust and modify them once they are created.

Participants also frequently provided additional information to support their labels, such as comments or specific parameters that led them to create the annotation. This contextual information could be used as a surrogate for the thought process of the annotators and further inform the importance of specific parameters on the target label. The digital tool should therefore incorporate the ability to provide additional data, such as parameters of interest, confidence in the accuracy of created labels, and free-form text that provides further context.

Finally, the feedback sought upon completing the annotation indicated the need for both a progress indicator and the importance of preservation of the continuity of the annotation process on retaining participants’ focus. A digital tool could account for this by displaying the count and information about the already created labels and making it easy and efficient for participants to annotate consecutive admissions. As such, the functional requirements for the digital data annotation tool can be specified as follows:

- R1: Data analysis in which participants are free to navigate through the entire span of the admission data, as well as underlying patient demographics.
- R2: Label creation functionality which is flexible enough to allow end users to select the start and end of annotation independently and in any order.
- R3: Label adjustment capability that facilitates an easy and convenient way to amend the created label, particularly with that label presented on top of data.
- R4: Label supplementation feature that allows participants to provide additional context for the created annotation, including confidence in the label accuracy and relevance of different parameters.
- R5: Workflow continuity that ensures a cohesive process of annotation and keeps the end users engaged and focused on the task.

#### Semiautomated Annotation

Facilitating a semiautomated approach to annotation that allows for creating labels for an entire dataset is a separate but closely related task. As such, it can benefit from the analysis of the manual annotation process and use it to further inform the requirements for the digital tool. The annotation of a single admission could be described as a bottom-up approach, in which end users are presented with data specific to a single admission and asked to annotate them directly (optimally until the entire dataset is annotated). Conversely, adopting an approach that automates the process would likely involve the annotation of an entire dataset based on a single user input, constituting a top-down approach. A semiautomated approach would therefore focus on creating labels for the whole dataset without focusing on individual admissions during their creation but potentially using them to evaluate the annotations and refine them upon further analysis. To that extent, the digital tool needs to allow its end users to establish annotation strategies that are independent of individual admissions during their formulation.

The process in which participants interacted with the individual admissions suggested several key requirements that needed to be adapted for the semiautomated approach. To close the loop of investigating, annotating, evaluating, and refining that the participants exhibited during the simulated activity, the semiautomated annotation approach facilitated by the platform should also allow for analysis of the effectiveness of the annotation in the context of both the entire dataset and individual admissions. In particular, the interface should allow the end users to view individual admissions with the created annotations applied, providing additional explanations for why the label was created, and in turn, allowing them to refine their annotation strategies. Consequently, the requirements for the digital tool facilitating a semiautomated annotation are defined as follows:

- R6: Annotation and evaluation loop captured as one continuous process that preserves the focus of the end users and facilitates effective annotation.
- R7: Label analysis of individual admissions with the overlay of created annotations on the time-series data and justification for their presence.
- R8: Dataset-wide performance metrics that capture the effectiveness of the annotation in the form of aggregate statistics computed across the entire dataset.

#### Operational Constraints

In addition to the requirements identified through the analysis of participants’ approach to the annotation task, there are further operational constraints that need to be reconciled to deliver an effective annotation workflow. To accommodate the busy schedules of the ICU staff, the tool should allow its end users to access it in a way that suits their needs and does not impose strict time commitments. As such, end users should be able to perform the annotation asynchronously and access the platform remotely. Furthermore, to preserve the focus and ensure that the end users’ time is used efficiently, the system should provide a responsive and time-efficient workflow that enables them to create and evaluate annotations in a short span of time. This is particularly important for the semiautomated approach in which the timely annotation of a large volume of data is critical to preserve the continuity of the annotation and evaluation loop. The operational requirements for the annotation tool are therefore defined as follows:

- R9: Asynchronous and remote annotation that allows the end users to perform the task at their convenience without further complicating their busy schedules.
- R10: Responsiveness of the system that provides feedback on the created annotations in a timely manner, particularly in the case of the semiautomated annotation.

#### Labels for ML

Finally, having identified the functional and operational requirements, we analyzed the platform in the wider context of using the created labels in ML workflows. To that extent, the discussion between data science experts within our research team surfaced several key factors that the tool should account for to ensure that the captured data can seamlessly integrate into ML pipelines.

The most important one to consider is the need to achieve a careful balance between the number of annotated admissions and the confidence in the created annotations. On one hand, increasing the total number of annotated admissions and their diversity would result in a larger training dataset and, consequently, improve the performance of the resulting ML model. On the other hand, to account for the biases of individual annotators, there needs to be some overlap of admissions annotated by distinct annotators that would allow for a comparison of their approaches and biases. Furthermore, for this to happen, additional metadata surrounding the confidence in the specific annotations and relating the created annotations to their authors also needs to be collected. Only then, will the data scientists be able to evaluate each of the annotators individually, compute their biases, and adjust their annotations accordingly to improve the robustness of the training dataset.

Depending on the volume of the data that needs to be annotated, the nature of the annotation task, and the number and experience of the annotators, this balance might be substantially different. Because of this, the system needs to facilitate a dynamic and adjustable configuration for how specific data are assigned to the annotators. This suggests that in addition to the previously established requirements, the tool should also aim to satisfy the following ML-specific requirement:

- R11: Flexible data-splitting solution that allows for adjustment of the data assigned to each participant and keeps track of the label authorship.

## Discussion

### Principal Results

This study aimed to explore how intensive care experts approach data annotation to establish requirements for a digital tool designed for annotating large datasets in intensive care. We conducted a manual data annotation activity, observing participants as they completed the task and analyzing their actions to gain insights into their annotation strategies. This analysis informed the development of software requirements for a digital data annotation platform.

Our findings revealed that the time-series annotation process follows a cyclical annotation-evaluation loop, which includes data investigation, label creation, reevaluation of data with the labels applied, and refinement of the created labels. From this analysis, we constructed 11 key requirements: 5 directly related to facilitating the annotation of individual patient admissions, 3 adapted for implementing a semiautomated annotation feature, 2 operational requirements focused on the effectiveness of the annotation workflow, and 1 requirement addressing the future use of labels in the ML context (Table 1).

**Table 1:** Requirements generated from analysis of observations made during simulated annotation activity.

Category		Requirement description
**Annotation of individual admissions**		
	R1	Data analysis in which participants are free to navigate through the entire span of the admission data, as well as underlying patient demographics.
	R2	Label creation functionality which is flexible enough to allow end users to select the start and end of annotation independently and in any order.
	R3	Label adjustment capability that facilitates an easy and convenient way to amend the created label, particularly with that label presented on top of data.
	R4	Label supplementation feature that allows participants to provide additional context for the created annotation, including confidence in the label accuracy and relevance of different parameters.
	R5	Workflow continuity that ensures a cohesive process of annotation and keeps the end users engaged and focused on the task.
**Semiautomated annotation**		
	R6	Annotation and evaluation loop captured as one continuous process that preserves the focus of the end users and facilitates effective annotation.
	R7	Label analysis of individual admissions with the overlay of created annotations on the time-series data and justification for their presence.
	R8	Dataset-wide performance metrics that capture the effectiveness of the annotation in the form of aggregate statistics computed across the entire dataset.
**Operational constraints**		
	R9	Asynchronous and remote annotation that allows the end users to perform the task at their convenience without further complicating their busy schedules.
	R10	Responsiveness of the system that provides feedback on the created annotations in a timely manner, particularly in the case of the semiautomated annotation.
**Use in machine learning**		
	R11	Flexible data-splitting solution that allows for adjustment of the data assigned to each participant and keeps track of the label authorship.

#### Approach to Annotation

The observations made during the manual annotation activity highlighted important characteristics of how experts from a clinical background approach the annotation task. While the sample of our study produced no discernable differences in how participants from different roles approached the task of data annotation, we observed a variety of strategies used to complete the task. These included several techniques for initial familiarization with the data, prioritization of different parameters during the investigation process, and different order of operations when creating the label. Crucially, we observed that irrespective of the specific strategies, annotators followed a cyclic process of investigation, annotation, reevaluation, and refinement when annotating data. By modeling the process using NIC, we were able to analyze the observations made during the task completion stage and formulate the requirements for the software interface of a digital data annotation platform.

In our analysis, we found that the nature of how annotators choose to analyze the data is largely preferential and, therefore, requires a degree of flexibility in how the software presents the data and allows its users to navigate through it. The cyclic nature of the process suggested that to ensure effective annotation, it must facilitate a continuous and intuitive loop of annotation, allow users to perform actions in varying order, and seamlessly adapt to different stages of the process. Since these requirements were derived from the simulated annotation of individual admissions but captured the characteristics of the overall problem of data annotation, the requirements for the semiautomated approach were adapted to ensure the continuity of the annotation-evaluation loop and flexibility in analysis.

Finally, the fact that annotators frequently sought feedback after creating labels further emphasized the importance of ensuring a seamless and uninterrupted annotation workflow and suggested the need to integrate progress indicators within the digital platform. Together with individual preferences for focusing on different parameters during the analysis and supplying additional information as part of the created labels, this also highlighted the need to capture contextual information alongside labels, which could be beneficial for their later use in the ML pipelines.

#### Design Implications

This literature on involving clinical staff in the collaborative design of software interventions shows that it can have profound effects on user acceptance and uptake upon release [23,39]. However, it is important to highlight that effectively engaging in such collaboration can be a challenging task, particularly when research activities require substantial time commitment [40]. Collaborating with intensive care clinicians, whose time is in high demand and short supply, may therefore require additional accommodations to be effective. In the context of annotating data, these accommodations translate into additional requirements that adapt the process to the busy schedules of the clinical staff by allowing them to annotate data both asynchronously and remotely. This further reinforces the need to ensure that the software delivers an intuitive and time-efficient annotation workflow and places additional constraints on its design. Specifically, to facilitate the responsiveness of the annotation-evaluation loop in the semiautomated approach, where users can frequently simultaneously annotate the entire dataset, the software should incorporate a solution that dynamically adapts to both the number of active users and the volume of data that needs to be processed.

### Limitations and Future Work

The activity focused on capturing the approach to data annotation was conducted with a limited number of participants, which resulted in a limited diversity of clinical roles and, potentially, perspectives from the ICU. This suggests that some of the requirements established in this research could be particularly applicable to annotators in junior doctor positions and therefore not necessarily generalizable across entire staff working in ICUs. The selection of the task for the manual annotation activity was made to provide an annotation experience that could be extrapolated beyond the specifics of the task. Despite this, we acknowledge that the results it produced could be biased specifically toward the annotation of weaning from mechanical ventilation.

Furthermore, our approach to capturing the annotation process during the simulated activity with only a single observer had a significant impact on the quantity and quality of observations that were collected. Due to the imbalance in the number of annotators and observers, some of the actions undertaken by the participants during the annotation could have gone unnoticed. Furthermore, the presence of a single observer created an inherent bias in the collected observations, as the observed actions could have been perceived differently by observers with different backgrounds or characteristics. These limitations suggest that the observations collected during the activity come with a degree of incompleteness and inaccuracy, which may have impacted the elicited requirements.

We acknowledge that further work in establishing the requirements for the digital tool could strengthen the understanding of how clinicians approach the data annotation task. To that extent, we suggest that further research in this area focuses on capturing the requirements that expand beyond the confines of a single annotation task and within a broader and more diverse population from the ICUs. Capturing a wider range of perspectives could inform the applicability of the elicited requirements and, in consequence, strengthen the resulting design of the digital tool.

The nature of the activity itself was focused strictly on the direct annotation of individual admissions rather than the use of any assistive or automated technologies. Although several requirements elicited in this context applied to the overall annotation process, including a semiautomated approach, additional requirements not captured in this study may also exist. Further research should therefore focus on the evaluation of the proposed requirements and their use and limitations in real-world applications. Therefore, these requirements should be used to design and implement a digital data annotation platform that should be trialed within a clinical setting. Conducting a study that investigates the feasibility of a semiautomated approach to the data annotation, particularly in comparison with the direct annotation of individual admissions, could further inform the requirements for data annotation tools.

### Conclusions

In this study, we investigated how clinical staff from ICUs approach the task of data annotation and established 11 key requirements for a digital data annotation tool that could be deployed within the health care setting. Our findings revealed that data annotation is a cyclic process that demands flexibility in how annotators investigate and annotate the data. Preservation of the workflow continuity across different admissions and fluid transition between analysis, annotation, and refinement of the label are essential to facilitating effective data annotation in the clinical domain. Adaptations for the semiautomated annotation need to consider these factors by providing a responsive interface that dynamically adapts to the volume of data and allows for analysis on both an individual and a dataset-wide basis. The significance of these findings is evident in their potential to guide the development of a data annotation tool that capitalizes on the considerable data generated in the ICUs and the expanding use of computational methods in health care.

## Abbreviations

AI: artificial intelligence
ICU: intensive care unit
ML: machine learning
NIC: Norman’s Interaction Cycle
